# 伴t(14;18)(q32;q21)的慢性淋巴细胞白血病八例报告并文献复习

**DOI:** 10.3760/cma.j.issn.0253-2727.2021.07.008

**Published:** 2021-07

**Authors:** 菁 张, 海荣 仇, 慧 杨, 睿 郭, 祎 缪, 华渊 朱, 莉 王, 磊 范, 卫 徐, 建勇 李

**Affiliations:** 南京医科大学第一附属医院，江苏省人民医院血液科 210029 Department of Hematology, Jiangsu Province Hospital, The First Affiliated Hospital of Nanjing Medical University, Nanjing 210029, China

**Keywords:** 白血病，淋巴细胞，慢性, t(14;18)(q32;q21), 临床特征, 预后, Leukemia, lymphocytic, chronic, t(14;18)(q32;q21), Clinical features, Prognosis

## Abstract

**目的:**

分析伴t(14;18)(q32;q21)的慢性淋巴细胞白血病（CLL）患者的临床特征及预后，并进行相关文献复习。

**方法:**

收集并分析2009年11月至2019年11月于江苏省人民医院就诊的8例伴t(14;18)(q32;q21)的CLL患者的临床资料。

**结果:**

8例患者中7例男性，1例女性，诊断时中位年龄70岁，3例免疫表型积分5分，4例积分4分，1例积分3分。所有患者的骨髓组织病理学均为典型CLL表现。染色体核型示所有患者的t(14;18)(q32;q21)均为干系，3例仅携带t(14;18)(q32;q21)异常，4例为t(14;18)(q32;q21)伴+12，1例为t(14;18)(q32;q21)伴13q−。通过FISH在另外3例患者中发现了13q−。6例检测了免疫球蛋白重链可变区（IGHV）突变状态且均为有突变，未见IGHV3-21片段使用。进行相关检测的患者中，仅1例携带TP53突变，其余患者未见TP53、SF3B1、NOTCH1、MYD88突变。中位随访30.9个月时，1例死亡，7例存活，其中3例尚未达到治疗指征，4例接受化疗或免疫治疗的患者病情均稳定。

**结论:**

t(14;18)(q32;q21)在CLL中少见，往往与+12、有突变的IGHV伴随出现。伴t(14;18)(q32;q21)的CLL可能预后良好。

## Abstract (en)

**Objective:**

The study aimed to analyze the clinical features and prognosis of chronic lymphocytic leukemia (CLL) with t(14;18)(q32;q21) and conduct a literature review.

**Methods:**

The clinical data of 8 patients with CLL carrying t(14;18)(q32;q21) seen in Jiangsu Province Hospital from November 2009 to November 2019 were collected and analyzed.

**Results:**

Among the 8 cases, 7 were male and 1 was female. The median age at diagnosis was 70 years old. The immunophenotype score was 5 in 3 patients. 4 patients were scored 4 and the remaining one scored 3. The bone marrow histopathology showed the typical manifestation of CLL. Karyotype analysis showed that all the cases carried t(14;18)(q32;q21) in the stemline. The t(14;18)(q32;q21) showed as the sole abnormality in 3 cases, with +12 in 4, and with 13q− in 1 case. 13q− was found in another 3 patients by FISH. Immunoglobulin heavy chain gene (IGHV) mutation status was detected in 6 cases and all of them were mutated. None of them used IGHV3-21. Only 1 case harbored TP53 mutation and no TP53, SF3B1, NOTCH1, or MYD88 mutations were found in the remaining cases who underwent the relevant tests. At a median follow-up of 30.9 months, 1 case died. The remaining 7 cases survived and 3 of them have not reached the treatment indication. 4 patients who received chemotherapy or immunotherapy were stable.

**Conclusion:**

The t(14;18)(q32;q21) is rare in CLL and often accompanied by +12 and mutated IGHV. CLL with t(14;18)(q32;q21) tends to have a good prognosis.

慢性淋巴细胞白血病（CLL）是一种成熟B淋巴细胞克隆增殖性肿瘤，以淋巴细胞在外周血、骨髓、脾脏和淋巴结聚集为特征，其临床异质性较强。在众多预后因素中，细胞遗传学异常提供了重要的预后信息。随着CpG寡脱氧核苷酸（CpG-ODN）联合IL-2刺激方法的应用，染色体核型分析的成功率和异常率分别可达90％和80％[1]。染色体易位在所有CLL中的发生率为19.8％～34.0％，涉及IGH基因的14q32易位是其中较为常见的类型，其伙伴基因多为BCL2/18q21、BCL3/19q13、MYC/8q24[2]–[6]。国内关于t(14;18)(q32;q21)在CLL中的报道罕见，本文报道我院收治的8例伴t(14;18)(q32;q21)的CLL患者，并对相关文献进行复习。

## 病例与方法

1. 病例：回顾性分析了2009年11月至2019年11月于江苏省人民医院行CpG-ODN联合IL-2刺激染色体核型分析的CLL患者，在初治且染色体核型分析成功（定义为≥20个正常核型分裂象或有克隆性异常）的272例患者中，伴t(14;18)(q32;q21)者8例（2.9％），其中1例既往已报道[7]。8例患者中有7例于我院确诊，1例于外院确诊后至我院随访。收集8例患者的临床资料，包括性别、诊断时年龄、Rai和Binet分期、症状及体征、实验室检查、免疫表型、骨髓组织病理学、细胞遗传学及分子特征、治疗指征、治疗方案及疗效评估、生存时间及生存状态。CLL的诊断及治疗标准参照中国慢性淋巴细胞白血病工作组（cwCLL）2018版CLL诊疗指南[8]。

2. 随访：通过查阅门诊及住院病历或电话进行随访，随访截至2019年12月20日，中位随访时间30.9（8.7～71.6）个月。无治疗生存（TFS）期定义为自诊断到开始接受治疗或随访终点的时间间隔。总生存（OS）期定义为自诊断至任何原因导致死亡或随访终点的时间间隔。

## 结果

1. 临床特征：8例患者中，7例为男性，1例为女性。诊断时中位年龄为70（47～80）岁，2例为早期（Binet A/Rai 0或1），3例为中期（Binet B/Rai 2），3例为晚期（Binet C/Rai 3或4）。1例患者有疲乏及盗汗症状，3例有淋巴结肿大，2例有脾肿大，所有患者均未见肝肿大。中位外周血淋巴细胞绝对计数为29.91（9.53～103.24）×10^9^/L，1例HGB<100 g/L，2例PLT<100×10^9^/L。除1例患者数据缺失外，7例患者LDH平均值<正常值上限（271 U/L），6例患者检测了β_2_-微球蛋白水平，其中有4例β_2_-微球蛋白>3.5 mg/L（表1）。

**表1:** 8例伴t(14;18)(q32;q21)的慢性淋巴细胞白血病患者临床特征

例号	性别	年龄（岁）	Rai分期	Binet分期	B症状	淋巴结肿大	脾肿大	肝肿大	ALC（×10^9^/L）	HGB（g/L）	PLT（×10^9^/L）	LDH（U/L）	β_2_-MG（mg/L）
1	男	80	0	A	无	无	无	无	15.83	134	100	179	3.58
2	男	74	3	C	无	无	无	无	42.01	84	166	230	13.90
3	男	69	2	A	无	无	有	无	9.53	140	132	263	4.31
4	男	62	2	B	无	有	有	无	103.24	127	203	182	NA
5	男	47	1	B	疲乏及盗汗	有	无	无	42.12	133	151	225	2.90
6	男	71	4	C	无	无	无	无	17.80	148	94	NA	NA
7	男	72	4	C	无	无	无	无	9.87	138	70	210	15.90
8	女	65	1	A	无	有	无	无	53.57	111	110	216	3.32

注：B症状：不明原因的发热（体温38 °C以上）、盗汗、体重减轻（6个月内不明原因体重减轻10％以上）；ALC：淋巴细胞绝对计数；β_2_-MG：β_2_-微球蛋白；NA：未检测或数据缺失

2. 免疫表型及组织病理学：8例患者均进行了外周血流式细胞术免疫表型检测，3例积分5分。4例积分4分，其中1例为CD22强表达，3例为表面免疫球蛋白（sIg）强表达。1例积分3分（例6），表现为CD23弱表达和CD22强表达。所有患者均为CD10阴性。除1例患者数据缺失外，所有患者均为CD200强表达，CD103阴性，sIgM阴性或弱表达。8例患者中有2例CD38>30％。4例检测ZAP70的患者中3例为阴性，1例>20％。6例检测CD49d的患者中5例为阴性，1例>30％（表2）。为排除套细胞淋巴瘤，通过FISH检测了例6的IGH/CCND1易位，结果为阴性。

**表2:** 8例伴t(14;18)(q32;q21)的慢性淋巴细胞白血病患者免疫表型

例号	抗原表达率（％）													积分
	CD5	CD23	FMC7	Kappa	Lambda	CD22	CD200	CD103	CD10	sIgM	CD38	ZAP70	CD49d	
1	72.4	57.4	阴性	49.0	阴性	79.9	83.2	阴性	阴性	阴性	阴性	NA	阴性	4
2	58.1	70.5	阴性	78.0	阴性	阴性	92.0	阴性	阴性	阴性	阴性	阴性	阴性	4
3	51.0	43.0	阴性	45.0	阴性	30.0	80.3	阴性	阴性	阴性	阴性	NA	阴性	5
4	96.0	60.4	阴性	98.0	阴性	阴性	96.0	阴性	阴性	48.7	10.0	25.0	NA	4
5	65.0	40.0	阴性	阴性	98.0	阴性	85.0	阴性	阴性	30.0	93.0	阴性	阴性	4
6	45.0	29.0	阴性	阴性	52.0	95.0	92.0	阴性	阴性	阴性	92.0	阴性	95.0	3
7^a^	阳性	阳性	阴性	阴性	弱表达	弱表达	强表达	阴性	阴性	阴性	阴性	NA	阴性	5
8^a^	阳性	阳性	阴性	弱表达	阴性	阴性	NA	NA	阴性	NA	阴性	NA	NA	5

注：^a^未提供抗原表达率具体数值；NA：未检测或数据缺失

所有患者均进行了骨髓组织病理学检查，可见淋巴细胞弥漫性或结节样增生，胞体小，胞质少，核圆或椭圆，核染色质粗，不见核仁，免疫组化为CD5（+）、CD10（−）、CD20（+）、CD23（+）、LEF1（+）、CyclinD1（−）、SOX11（−）。

8例患者中有2例行淋巴结活检，例4右颈部淋巴结活检病理示：肿瘤细胞CD5（+）、CD20（++）、PAX-5（++）、CD23（+）、BCL2（++）、CyclinD1（+）、CD3（−）、CD10（−）、CD38（−）、BCL6（−）。通过FISH检测淋巴结活检组织的IGH/CCND1基因，呈一红一绿一黄信号的比例约为5％，而骨髓标本IGH/CCND1融合基因FISH检测为阴性。例8的右颌下淋巴结活检病理示：肿瘤细胞CD20（+）、PAX5（+）、CD79a（+）、CD23（+）、BCL2（+）、CD5（−）、CD10（−）、BCL6（−）、CyclinD1（−）。

3. 遗传学及分子特征：所有患者均用外周血或骨髓标本行CpG-ODN联合IL-2刺激的染色体核型分析（表3）。7例患者在诊断时即携带t(14;18)(q32;q21)，其中例2、例8于治疗后复查了染色体，仍有t(14;18)(q32;q21)，此时这两例均为疾病进展状态。另外1例患者2014年于外院诊断时染色体示46，XY[14]（24 h培养未刺激），2018年因出现治疗指征于我院就诊，行染色体核型分析见t(14;18)(q32;q21)。所有患者的t(14;18)(q32;q21)均为干系。3例患者仅携带t(14;18)(q32;q21)异常，4例为t(14;18)(q32;q21)伴+12，1例为t(14;18)(q32;q21)伴13q−。除例1未行检测外，FISH显示所有患者的IGH重排阳性，另外在3例患者中发现了染色体核型未揭示的13q−（表3）。

**表3:** 8例伴t(14;18)(q32;q21)的慢性淋巴细胞白血病患者遗传学特征

例号	时间	染色体核型	FISH					
			IGH重排	+12	13q−	11q−	17p−	6q−
1	诊断时	46,XY,t(14;18)(q32;q21)[5]/47,idem,+12[3]/46,XY[2]	NA	NA	NA	NA	NA	NA
2	诊断时	46,XY,t(14;18)(q32;q21)[4]/46,XY[6]	阳性	阴性	阳性	阴性	阴性	阴性
	治疗后	46,XY,t(14;18)(q32;q21)[6]/46,XY[4]	NA	NA	NA	NA	NA	NA
3	诊断时	46,XY,t(14;18)(q32;q21)[8]/46,XY[2]	阳性	阴性	阳性	阴性	阴性	阴性
4	诊断时	46,XY,t(14;18)(q32;q21)[7]	阳性	阴性	阳性	阴性	阴性	阴性
5	诊断时	47,XY,+12,t(14;18)(q32;q21)[5]/47,X,-Y,+der(3)*2[1]/46,XY[4]	阳性	阳性	阴性	阴性	阴性	阴性
6	诊断时	46,XY[14]	NA	NA	NA	NA	NA	NA
	治疗前	47,XY,+12,t(14;18)(q32;q21)[4]/47,XY,+12[6]	阳性	阳性	阴性	阴性	阴性	阴性
7	诊断时	46,XY,del(13)(q12q22),t(14;18)(q32;q21)[7]/46,XY[3]	阳性	阴性	阳性	阴性	阴性	阴性
8	诊断时	47,XX,+12,t(14;18)(q32;q21)[7]/46,XX[3]	阳性	阳性	阴性	阴性	阴性	阴性
	治疗后	47,XX,+12,t(14;18)(q32;q21)[2]/46,XX[8]	NA	NA	NA	NA	NA	NA

注：NA：未检测或数据缺失

6例患者检测了免疫球蛋白重链可变区（IGHV）突变状态且均为突变阳性，符合率及片段使用见表4。在完善基因检测的例2、5、6、7、8患者中，例8携带TP53突变c.829T>G，其余患者TP53、SF3B1、NOTCH1、MYD88突变均为阴性。例4仅检测了TP53突变，结果为阴性（表4）。

**表4:** 8例伴t(14;18)(q32;q21)的慢性淋巴细胞白血病患者免疫球蛋白重链可变区（IGHV）及基因突变情况

例号	IGHV			基因突变			
	突变状态	符合率（％）	片段使用	TP53	SF3B1	NOTCH1	MYD88
1	有突变	90.97	3-30*04	NA	NA	NA	NA
2	有突变	94.56	3-49*04	阴性	阴性	阴性	阴性
3	NA	NA	NA	NA	NA	NA	NA
4	NA	NA	NA	阴性	NA	NA	NA
5	有突变	93.40	1-2*02	阴性	阴性	阴性	阴性
6	有突变	95.92	3-15*01	阴性	阴性	阴性	阴性
7	有突变	93.40	1-18*01	阴性	阴性	阴性	阴性
8	有突变	93.40	3-23*01	c.829T>G	阴性	阴性	阴性

注：NA：未检测或数据缺失

4. 治疗及转归：截至随访终点，8例患者中1例死亡，7例存活，其中例1、3、7尚未达到治疗指征，TFS期及OS期分别为13.9、34.9、8.7个月。例2因HGB进行性减少接受了利妥昔单抗（RTX）治疗，4个周期后疗效评估为疾病稳定（SD），后予RTX维持治疗1个周期。1年后例2因2个月内淋巴细胞增多>50％，接受RTX治疗4个周期，疗效评估SD。例2的TFS期为0个月，OS期为27.8个月。例4因2个月内淋巴细胞增多>50％接受了CHOP方案（环磷酰胺+阿霉素+长春新碱+泼尼松）治疗，3个周期后疗效评估SD，遂调整为FC方案（氟达拉滨+环磷酰胺）治疗3个周期，疗效评估为部分缓解。该患者TFS期为2.3个月，OS期为43.5个月。例5在诊断时即有疲乏、盗汗，后至外院行8个周期RTX治疗，疗效评估不详。例5的TFS期为0个月，OS期为34.1个月。例6于2014年在外院确诊，后定期随访，未予治疗，2018年因进行性血小板减少于我院接受了RTX及苯丁酸氮芥治疗，2个周期后因心血管系统疾病停药，后病情稳定。2019年11月例6因进行性体重下降再次口服苯丁酸氮芥。例6的TFS期为58.0个月，OS期为71.6个月。例8因夜间盗汗>1个月接受了苯达莫司汀治疗，6个周期后疗效评估为疾病进展，随后进行了3个周期RTX治疗，然而其疾病快速进展直至死亡。例8的TFS期为8.4个月，OS期为18.9个月。

## 讨论

涉及IGH/BCL2基因重排的t(14;18)(q32;q21)是滤泡性淋巴瘤的相对特异性遗传学标志，但在CLL中较为罕见。本中心初治CLL中t(14;18)(q32;q21)的发生率为2.9％，与国外报道的数据相近[9]。

IGH和BCL2位点的断裂分别与重组激活基因和活化诱导胞苷脱氨酶参与的事件相关，两类事件共同介导了t(14;18)(q32;q21)的形成[10]。既往的病例报道表明，t(14;18)(q32;q21)异常主要为干系克隆[6],[11]–[12]。同样地，我们报道的8例CLL患者的t(14;18)(q32;q21)均为干系，且其中7例诊断时即携带该异常，这可能表明t(14;18)(q32;q21)是CLL发病过程中主要的早期遗传学事件之一。曾有假说提出t(14;18)(q32;q21)的存在与CLL起源于生发中心B细胞有关[13]，然而Baseggio等[14]通过检测BCL6和FAS基因突变否定了该假说。另外1例患者诊断时的染色体核型未见t(14;18)(q32;q21)，但在出现治疗指征的同时发现了该异常，可能是由于两次检测方法不同，CpG-ODN联合IL-2刺激方法能够提高染色体异常的检出率。但鉴于CLL中存在较频繁的克隆演变[15]，此现象也不排除是染色体的动态变化，如能被进一步证实，可能意味着t(14;18)(q32;q21)与CLL的疾病进展有一定联系。

+12是CLL中第二常见的遗传学异常，阳性率近20％，且与不典型免疫表型有关，如强表达的CD19、CD22、CD20、CD79b、CD38、CD49d、sIg和弱表达的CD43[16]。既往研究提出，伴t(14;18)(q32;q21)的CLL中+12常见，其阳性率约35％～50％，明显高于其他CLL群体[11],[14],[17]–[18]。我们的报道也证实了这一现象，在8例患者中有4例为t(14;18)(q32;q21)伴+12，其中3例积分为3～4分，不典型之处表现为强表达的CD22、sIg及弱表达的CD23。然而，2例仅携带t(14;18)(q32;q21)异常的患者均为sIg强表达，这可能提示t(14;18)(q32;q21)与不典型的免疫表型有一定联系，Geisler等[19]也报道了1例类似病例。

IGHV突变状态是CLL的重要独立预后因素，无突变的IGHV与较差的预后相关[20]。中国CLL患者的IGHV突变率约为60％，最常见的片段使用为IGHV4-34、IGHV3-23及IGHV3-7[21]。无论突变状态如何，使用IGHV3-21的患者预后较差[22]。已有文献提出伴t(14;18)(q32;q21)的CLL患者多携带有突变的IGHV，其频率约90％，高于其他CLL群体[11],[14],[17]。我们报道的结果也支持这一结论，8例患者中有6例检测了IGHV突变状态且均为有突变，未见IGHV3-21片段使用。值得注意的是，4例携带t(14;18)(q32;q21)伴+12的患者均为IGHV有突变状态，而+12患者中多见无突变的IGHV[23]，进一步说明了t(14;18)(q32;q21)与突变阳性IGHV之间的联系。

t(14;18)(q32;q21)在CLL中的预后意义仍有争议。曾有研究指出伴IGH易位CLL患者的TFS期、OS期较预后良好组（仅13q−异常或正常核型）及中风险组（+12或1～2种染色体异常）缩短，且IGH易位是独立的不良预后因素[24]。Tang等[11]也认为伴t(14;18)(q32;q21)的CLL预后较差，其研究报道了12例患者，其中11例需要化疗，5例死亡。而另一些研究则认为t(14;18)(q32;q21)与临床或生物侵袭性特征无关，不影响预后[14],[17]。Davids等[25]进一步指出在携带IGH易位的CLL中，伴t(14;18)(q32;q21)的患者较不伴t(14;18)(q32;q21)的患者有更长的TFS期，其预后与13q−异常组相近。上述研究得出不同结论的原因如下：①样本量较少；②纳入了除CLL以外的其他B淋巴细胞增殖性疾病；③纳入了除t(14;18)(q32;q21)以外的其他染色体易位。迄今为止样本量最大的研究共纳入38例伴t(14;18)(q32;q21)的初诊CLL，结果显示该组患者的TFS期与OS期均与13q−异常组相近[9]。我们报道的8例患者中仅1例呈侵袭性病程且难治，其预后差的原因可能是TP53突变。另外7例患者中未见复杂核型、IGHV无突变状态、11q−、TP53缺失或突变等不良预后因素。截至随访终点，7例患者均存活，其中3例尚未达到治疗指征，其余接受化疗或免疫治疗的患者均病情稳定。综上，伴t(14;18)(q32;q21)的CLL患者可能预后良好。然而，鉴于伴t(14;18)(q32;q21)的CLL患者多携带有突变的IGHV，其预后较好不排除与IGHV相关。有研究报道，在IGHV突变的CLL患者中，携带染色体易位的患者TFS期缩短[4]，因此t(14;18)(q32;q21)在CLL预后中的意义有待进一步确认。

综上所述，t(14;18)(q32;q21)在CLL中罕见，但有独特的临床及预后特征。伴t(14;18)(q32;q21)的CLL患者中+12、IGHV突变较常见。目前倾向于认为伴t(14;18)(q32;q21)的CLL患者预后与伴13q−异常患者相当，然而其确切的预后价值有待大型队列研究进一步证实。
